# Clinical Characteristics and Outcomes of Laboratory-Confirmed SARS-CoV-2 Cases Infected With Omicron Subvariants and the XBB Recombinant Variant

**DOI:** 10.7759/cureus.35261

**Published:** 2023-02-21

**Authors:** Rajesh P Karyakarte, Rashmita Das, Sonali Dudhate, Jeanne Agarasen, Praveena Pillai, Priyanka M Chandankhede, Rutika S Labhshetwar, Yogita Gadiyal, Mansi V Rajmane, Preeti P Kulkarni, Safanah Nizarudeen, Suvarna Joshi, Varsha Potdar, Krishanpal Karmodiya

**Affiliations:** 1 Microbiology, Byramjee Jeejeebhoy Government Medical College & Sassoon General Hospitals, Pune, IND; 2 Microbiology, Byramjee Jeejeebhoy Government Medical College, Pune, IND; 3 Infectious Disease, Indian Council of Medical Research-National Institute of Virology, Pune, IND; 4 Pediatrics, Byramjee Jeejeebhoy Government Medical College, Pune, IND; 5 Biology, Indian Institute of Science Education and Research, Pune, IND

**Keywords:** covid-19, xbb.1.5, xbb*, bq.1*, ba.5*, ba.2.75*, ba.2.38*, ba.2.10*, sars-cov-2

## Abstract

Background

SARS-CoV-2 has evolved to produce new variants causing successive waves of infection. Currently, six variants are being monitored by the World Health Organization that are replacing BA.5. These include BF.7 (BA.5 + R346T in spike), BQ.1 (and BQ.1.1, with BA.5 + R346T, K444T, N460K mutations in spike), BA.2.75 (including BA.2.75.2 and CH.1.1), and XBB (including XBB.1.5). BQ.1 and XBB variants are more immune evasive and have spread quickly throughout the world. Concerning the potential severity of infections caused by these variants, the present study describes the clinical characteristics and outcomes of these major variants in Maharashtra.

Methodology

A total of 1,141 reverse transcriptase-polymerase chain reaction (RT-PCR)-positive SARS-CoV-2 samples, with a cycle threshold (Ct) value of less than 25, were processed for SARS-CoV-2 whole genome sequencing between July 10, 2022, and January 12, 2023. All corresponding demographic and clinical data were recorded and analyzed using Microsoft® Excel and Epi Info™.

Results

Out of the 1,141 samples sequenced, BA.2.75* (63.78%) was the predominant Omicron variant, followed by the XBB* (18.88%), BA.2.38* (4.94%), BA.5* (4.06%), BA.2.10* (3.51%), and BQ.1* (1.65%). A total of 540 cases were contacted telephonically, of whom 494 (91.48%) were symptomatic with mild symptoms. Fever (77.73%) was the most common symptom, followed by cold (47.98%), cough (42.31%), and myalgia and fatigue (18.83%). Of the 540 cases, 414 (76.67%) cases recovered at home, and 126 (23.33%) were institutionally quarantined/hospitalized. Among the home-isolated and hospitalized cases, 416 (99.76%) and 108 (87.80%), respectively, recovered with symptomatic treatment, while one (0.24%) and 15 (12.20%), respectively, succumbed to the disease. Out of the 540 cases, 491 (90.93%) were vaccinated with at least one dose of the COVID-19 vaccine, 41 (7.59%) were unvaccinated, and for eight (1.48%) cases, vaccination data was not available.

Conclusions

The current study indicates that the XBB* variant is causing mild disease in India. However, as XBB* possesses both immune-escape and infectivity-enhancing mutations, it has the potential to spread to other parts of the world rapidly. Further, anti-SARS-CoV-2 vaccination improves survival rates in COVID-19.

## Introduction

SARS-CoV-2 has evolved continuously to give rise to new variants and drive successive waves of infection [1]. Since its first emergence in November 2021, the Omicron variant of concern (VOC) has become the most widespread and dominant variant globally. Currently, BA.5 and its descendant lineages are the dominant variants, followed by BA.2.75 and its subvariants (as of January 1, 2023) [2]. With a swarm of variants emerging and competing in the *variant soup* [3], there are four subvariants that are replacing the BA.5 descendent lineages and are being monitored by the World Health Organization [2]. These include BF.7 (BA.5 + R346T in spike), BQ.1 (and BQ.1.1, with BA.5 + R346T, K444T, N460K mutations in spike), BA.2.75 (including BA.2.75.2 and CH.1.1), and XBB (including XBB.1.5) [2]. BF.7, a sub-lineage of BA.5, was first identified in Belgium in May 2022 [4]. BQ.1, also a sub-lineage of BA.5, was first identified in Nigeria in early July 2022. There are 80 Pango lineages associated with the BQ.1* variant (* indicates the lineage and its sub-lineages), which have spread dramatically to Europe, South America, North America, and Africa [5,6]. XBB is a recombinant of BA.2.10.1 (first identified in April 2022 with a majority of sequences from India at the time of its naming [7]) and BA.2.75 sub-lineages, i.e., BJ.1 and BM.1.1.1, respectively, with a breakpoint in S1. It was first identified in mid-August 2022 and is suggested to have emerged around the Indian subcontinent [6]. XBB and its sub-lineages have spread quickly and have become dominant in India, Bangladesh, Malaysia, Singapore, and other parts of Asia [6,8]. Recently, a subvariant of XBB.1, the XBB.1.5 variant, has rapidly spread and has been detected in at least 46 countries and 49 states in the United States [9]. As these variants continue to evolve and diversify, they are of particular interest, as they are more immune evasive and expand rapidly due to additional mutations in their spike protein. There is evidence that these SARS-CoV-2 variants may further reduce the effectiveness of current COVID-19 vaccines and monoclonal antibody treatments [6].

For an effective pandemic response, it is essential to understand the range of illnesses associated with these new strains. There are concerns about the potential severity of infections caused by these variants due to multiple mutations in their spike protein, which may affect their ability to enter cells and escape the immune system [10]. Therefore, the present study describes the clinical characteristics and outcomes of the major variants identified during the community surveillance of SARS-CoV-2 in Maharashtra.

This article was previously posted to the medRxiv preprint server on January 6, 2023. The revised article was posted on January 27, 2023.

## Materials and methods

This study was conducted as part of the Indian SARS-CoV-2 Genomics Consortium (INSACOG) sequencing activity in Maharashtra to monitor genomic variations in the virus and study its epidemiological trends. The study protocol for SARS-CoV-2 whole genome sequencing was reviewed and approved by the Institutional Ethics Committee at Byramjee Jeejeebhoy Government Medical College (BJGMC), Pune, Indian Council of Medical Research-National Institute of Virology (ICMR-NIV), Pune, and Indian Institute of Science, Education and Research (IISER), Pune.

Sample acquisition

Samples from several reverse transcriptase-polymerase chain reaction (RT-PCR) swab collection and testing centers in various districts of Maharashtra and its neighboring states were sent to BJGMC, ICMR-NIV, and IISER, Pune, for whole genome sequencing.

Respiratory specimens, including nasopharyngeal and oropharyngeal swabs, positive for SARS-CoV-2 infection, were collected in viral transport media (VTM). These samples were transported to the sequencing laboratory in triple packaging, maintaining the cold chain, and stored at -80°C. According to government instructions and INSACOG, 5% of the samples from positive cases with a cycle threshold (Ct) value of less than 25, including clusters, vaccine breakthrough infections, cases with mild and moderate symptoms, and hospitalized and deceased cases, were processed.

RNA extraction, library preparation, next-generation sequencing, and lineage analysis

Total RNA was extracted from respiratory specimens using the MagMax™ viral/pathogen nucleic acid extraction kit (ThermoFisher Scientific, Waltham, MA, USA) on an automated extraction system, KingFisher Flex (ThermoFisher, Waltham, MA, USA), following the manufacturer’s instructions. Nucleic acid was eluted in 50 μL of elution buffer, and the RNA was quantified with the Qubit RNA High Sensitivity Kit using the Qubit® 2.0 Fluorometer (ThermoFisher Scientific, Waltham, MA, USA). This RNA was used for library preparation for sequencing.

Libraries for SARS-CoV-2 sequencing were prepared using the Rapid Barcoding Kit (RBK110.96) and Midnight RT-PCR Expansion Kits (EXP001) (BJGMC, Pune), Ion 540TM chip and the Ion Total RNA-Seq kit v2.0 (ThermoFisher Scientific, Waltham, MA, USA) (ICMR-NIV, Pune), and Illumina COVIDSeq RUO test kits (Illumina Inc, USA) (IISER, Pune). Sequencing was performed using GridION (ONT, Littlemore, United Kingdom), Ion S5 (ThermoFisher Scientific, Waltham, MA, USA), and the NextSeq 550 sequencing platform, respectively. Reads were aligned to the reference genome using the MinKNOW software, Iterative Refinement Meta-Assembler (IRMA), and the DRAGEN COVID Lineage application, respectively. Lineage analysis was done using Phylogenetic Assignment of Named Global Outbreak LINeages (PangoLIN) COVID-19 lineage assigner, version v4.1.3, pangolin-data version v1.17, and clade was analyzed using Nextclade software, version 2.9.1.

Demographic and clinical data collection

Demographic data, including the patient’s age, gender, area of residence, contact number, and date of specimen collection and testing, were collected from the centralized data entry portal for COVID-19 testing in India, the ICMR COVID-19 Data Portal using the unique identification number (ICMR ID). To gather additional information, telephonic interviews were conducted, and patients were interviewed individually. During the interview, the demographic details available on the portal were validated, and information on the presence of any symptoms at the time of testing, comorbidities, vaccination details, history of previous infections, hospitalization, oxygen requirement, and treatment was collected. Patients unwilling to share their history during the interview were excluded from the study.

Statistical analysis

All demographic and clinical data were recorded using Microsoft® Excel, and analysis was performed using Microsoft® Excel and Epi Info™, version 7.2.4.0. The continuous variables were presented as the median and interquartile range (IQR). The Kruskal-Wallis test was used to compare the median values between the variants. The categorical variables were presented as numbers and percentages. The chi-square test was used to compare categorical variables between the variants. Fisher’s exact test was used to compare the categorical values with limited data. A p-value <0.05 was considered statistically significant.

## Results

Demographic characteristics of the study population

**Figure 1:**
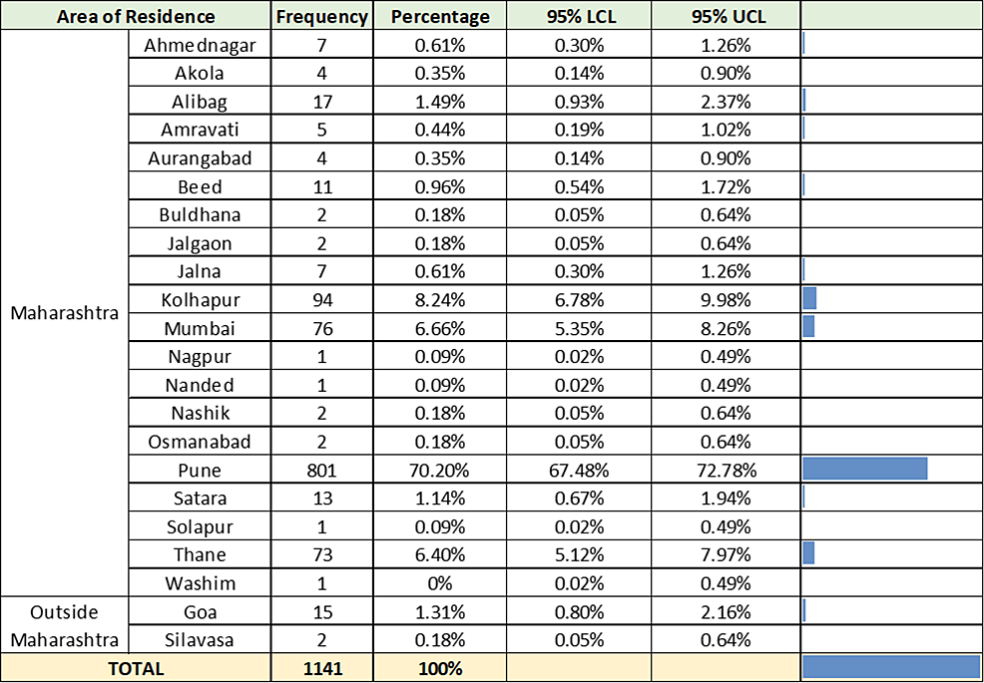
Geographical distribution of 1,141 RT-PCR-positive SARS-CoV-2 samples. RT-PCR = reverse transcriptase-polymerase chain reaction; UCL = upper confidence limit; LCL = lower confidence limit

Between July 10, 2022, and January 12, 2023, 1,141 RT-PCR-positive SARS-CoV-2 samples were collected and included in the study. Figure 1 describes the geographical distribution of the samples collected.

**Figure 2:**
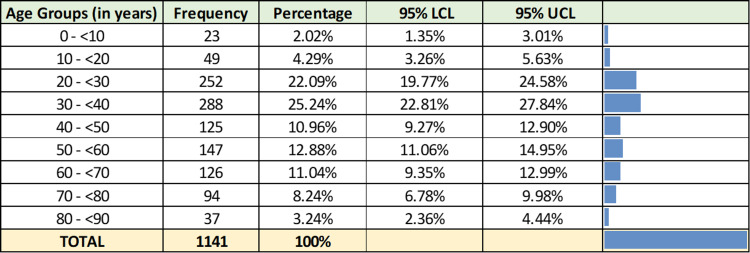
Age-wise distribution of 1,141 RT-PCR-positive SARS-CoV-2 samples. RT-PCR = reverse transcriptase-polymerase chain reaction; UCL = upper confidence limit; LCL = lower confidence limit

**Figure 3:**
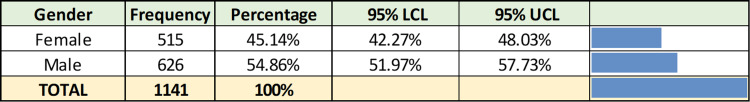
Gender-wise distribution of 1,141 RT-PCR-positive SARS-CoV-2 samples. RT-PCR = reverse transcriptase-polymerase chain reaction; UCL = upper confidence limit; LCL = lower confidence limit

The study population included cases from all age groups with a median age of 37 years (IQR = 28-57) (Figure 2). The male-to-female ratio was 1.22:1 (Figure 3).

SARS-CoV-2 lineage distribution in sequenced samples

Of the 1,141 samples sequenced, 911 (79.84%) were assigned Pango lineages, of which the BA.2.75* (63.78%) was the predominant Omicron variant, followed by the XBB* (18.88%), BA.2.38* (4.94%), BA.5* (4.06%), BA.2.10* (3.51%), and BQ.1* (1.65%) (Table 1).

**Table 1:** Variant distribution among the 1,141 SARS-CoV-2 samples sequenced.

Nextclade Clade assigned	Pangolin lineages assigned		Numbers (%)	
21K	B.1.1.529		01 (0.09%)	
21L	BA.2		12 (1.05%)	
21L	BA.2.10	BA.2.10*	01 (0.09%)	32 (2.80%)
	BA.2.10.1		26 (2.28%)	
	BA.2.10.4		04 (0.35%)	
	BJ.1		01 (0.09%)	
22C	BA.2.12.1		01 (0.09%)	
	BA.2.3.20		02 (0.18%)	
21L	BA.2.38	BA.2.38*	03 (0.26%)	45 (3.94%)
	BA.2.38.1		02 (0.18%)	
	BA.2.38.2		03 (0.26%)	
	BA.2.38.3		01 (0.09%)	
	BH.1		36 (3.16%)	
21L	BA.2.74		02 (0.18%)	
22D	BA.2.75	BA.2.75*	93 (8.15%)	581 (50.92%)
	BA.2.75.1		69 (6.05%)	
	BA.2.75.2		109 (9.55%)	
	BA.2.75.3		07 (0.61%)	
	BA.2.75.5		05 (0.44%)	
	BA.2.75.6		14 (1.23%)	
	BA.2.75.7		17 (1.49%)	
	BA.2.75.9		01 (0.09%)	
	BL.1		36 (3.16%)	
	BL.2		13 (1.14%)	
	BL.3		02 (0.18%)	
	BM.1		13 (1.14%)	
	BM.1.1		63 (5.52%)	
	BM.1.1.1		04 (0.35%)	
	BM.1.1.3		09 (0.79%)	
	BM.4.1		06 (0.53%)	
	BM.4.1.1		17 (1.49%)	
	BN.1		29 (2.54%)	
	BN.1.1		01 (0.09%)	
	BN.1.3		02 (0.18%)	
	BN.1.3.1		02 (0.18%)	
	BN.1.4		09 (0.79%)	
	BR.2.1		02 (0.18%)	
	BY.1		43 (3.77%)	
	CA.2		01 (0.09%)	
	CA.3		02 (0.18%)	
	CH.1		01 (0.09%)	
	CH.1.1		11 (0.96%)	
21L	BA.2.76		07 (0.61%)	
21L	BA.2.83		01 (0.09%)	
22B	BA.5	BA.5*	02 (0.18%)	37 (3.24%)
	BA.5.2		11 (0.96%)	
	BA.5.2.1		06 (0.53%)	
	BA.5.6		01 (0.09%)	
	BA.5.9		01 (0.09%)	
	BE.1.1		03 (0.26%)	
	BF.7		03 (0.26%)	
	BF.7.6		01 (0.09%)	
	BF.14		01 (0.09%)	
	BF.26		03 (0.26%)	
	BF.27		01 (0.09%)	
	BF.3		03 (0.26%)	
	BF.33		01 (0.09%)	
22E	BQ.1	BQ.1*	02 (0.18%)	15 (1.31%)
	BQ.1.1		05 (0.44%)	
	BQ.1.1.18		01 (0.09%)	
	BQ.1.1.22		01 (0.09%)	
	BQ.1.1.4		01 (0.09%)	
	BQ.1.2		02 (0.18%)	
	BQ.1.9		03 (0.26%)	
22F	XAR	Recombinant	01 (0.09%)	
	XBF		02 (0.18%)	
	XBB	XBB*	70 (6.13%)	172 (15.07%)
	XBB.1		41 (3.59%)	
	XBB.1.5		07 (0.61%)	
	XBB.2		31 (2.72%)	
	XBB.3		22 (1.93%)	
	XBB.5		01 (0.09%)	
Not assigned	Not assigned (QC failure)		230 (20.16%)	
Grand total			1141 (100%)	

**Figure 4:**
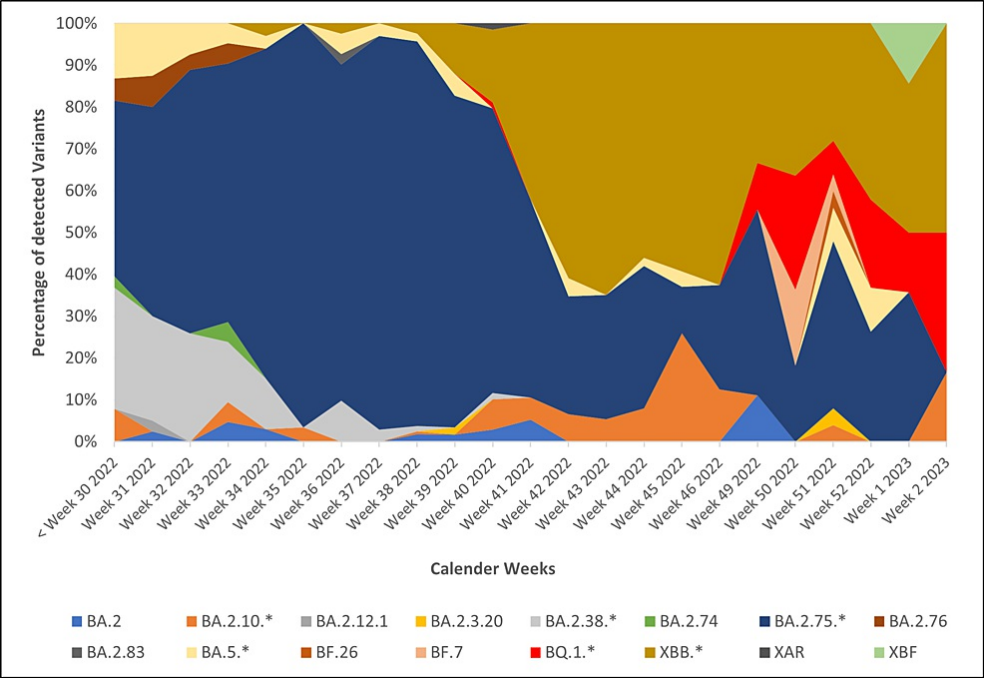
Temporal distribution of SARS-CoV-2 variants during the study period (from July 10, 2022, to January 12, 2023).

Figure 4 describes the temporal distribution of SARS-CoV-2 variants detected during the study period. The x-axis represents the calendar weeks, and the y-axis represents the percentage of each lineage from the sequenced samples.

Demographic and clinical characteristics of major SARS-CoV-2 variants detected during the study

Table 2 describes the demographic characteristics of the major SARS-CoV-2 variants detected in the study.

**Table 2:** Demographic characteristics of major SARS-CoV-2 variants detected during the study period (n = 882).

	BA.2.10*	BA.2.38*	BA.2.75*	BA.5*	BQ.1*	XBB*	Grand total
Gender-wise distribution, p-value = 0.088							
Male	22 (68.75%)	19 (42.22%)	311 (53.53%)	15 (40.54%)	10 (66.67%)	98 (56.98%)	475 (53.85%)
Female	10 (31.25%)	26 (57.78%)	270 (46.47%)	22 (59.46%)	05 (33.33%)	74 (43.02%)	407 (46.15%)
Median age, H (5) = 12.343, p-value = 0.030							
	29.5 (IQR: 20.5 – 43.0)	38.0 (IQR: 29.0 – 58.0)	38.0 (IQR: 28.0 – 57.0)	38.0 (IQR: 31.0 – 59.0)	55.0 (IQR: 41.0 – 71.0)	38.5 (IQR: 28.5 – 59.0)	
Age-wise distribution							
0 to <10	01 (3.13%)	00 (00%)	11 (1.89%)	01 (2.70%)	00 (00%)	03 (1.74%)	16 (1.81%)
10 to <20	07 (21.88%)	04 (8.89%)	25 (4.30%)	00 (00%)	00 (00%)	11 (6.40%)	47 (5.33%)
20 to <30	08 (25.00%)	08 (17.78%)	133 (22.89%)	06 (16.22%)	01 (6.67%)	32 (18.60%)	188 (21.32%)
30 to <40	04 (12.50%)	11 (24.44%)	140 (24.10%)	13 (35.14%)	02 (13.33%)	42 (24.42%)	212 (24.04%)
40 to <50	05 (15.63%)	06 (13.33%)	53 (9.12%)	03 (8.11%)	04 (26.67%)	21 (12.21%)	92 (10.43%)
50 to <60	01 (3.13%)	05 (11.11%)	86 (14.80%)	07 (18.92%)	02 (13.33%)	22 (12.79%)	123 (13.95%)
60 to <70	05 (15.63%)	04 (8.89%)	64 (11.02%)	05 (13.51%)	02 (13.33%)	18 (10.47%)	98 (11.11%)
70 to <80	01 (3.13%)	06 (13.33%)	48 (8.26%)	02 (5.41%)	03 (20.00%)	18 (10.47%)	78 (8.84%)
80 to <90	00 (00%)	01 (2.22%)	21 (3.61%)	00 (00%)	01 (6.67%)	05 (2.91%)	28 (3.17%)

**Figure 5:**
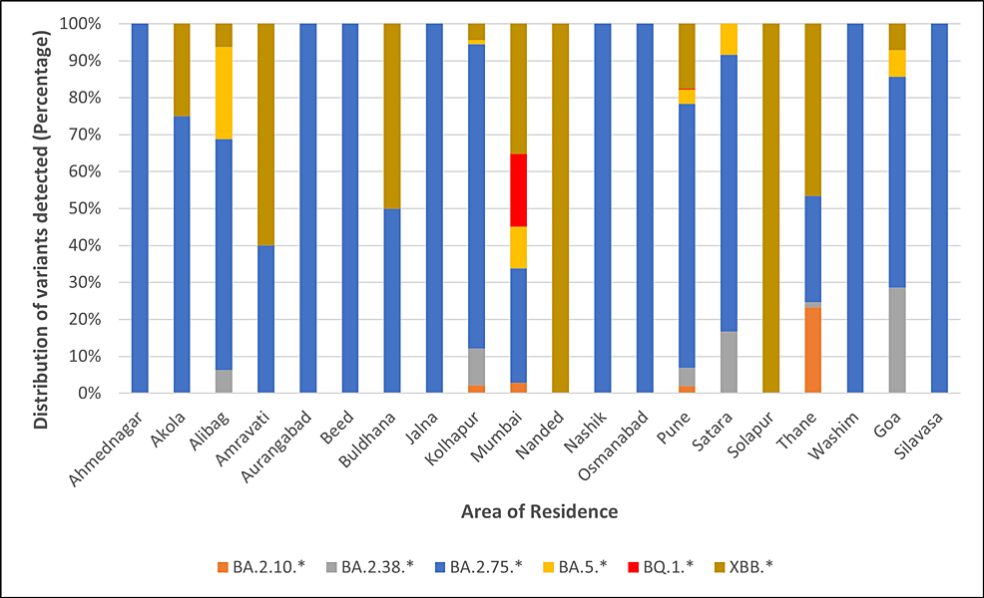
Distribution of major SARS-CoV-2 variants detected during the study versus the area of residence.

Out of 882 cases, there were 475 (53.85%) males and 407 (46.15%) females. The median age of cases infected with BA.2.10* was 29.5 years, BQ.1* was 55.0 years, XBB* was 38.5 years, and for BA.2.38*, BA.2.75*, and BA.5* variants, it was 38.0 years. There was a statistically significant difference in the median age (p = 0.030) of cases infected with different variants. Figure 5 depicts the distribution of major variants and the area of residence of cases.

Of the 882 cases, 540 (61.22%) could be telephonically contacted to obtain information regarding their symptoms, hospitalization, treatment, and vaccination status. Table 3 summarizes the clinical characteristics, vaccination status, and outcomes of 494 cases.

**Table 3:** Clinical characteristics and outcome of 540 patients infected with major variants (n = 540).

	BA.2.10*	BA.2.38*	BA.2.75*	BA.5*	BQ.1	XBB*	Grand total
Total number of cases with history available							
	17 (53.13%)	21 (46.67%)	374 (64.37%)	18 (48.65%)	10 (66.67%)	100 (58.14%)	540 (61.22%)
History of previous COVID-19 infection, p-value = 0.259							
	01 (5.88%)	03 (14.29%)	51 (13.64%)	00 (00%)	01 (10%)	15 (15%)	71 (13.15%)
Symptom status at the time of sample collection, p-value = 0.071							
Asymptomatic	03 (17.65%)	01 (4.76%)	28 (7.49%)	00 (00%)	03 (30%)	11 (11.%)	46 (8.52%)
Symptomatic	14 (82.35%)	20 (95.24%)	346 (92.51%)	18 (100%)	07 (70%)	89 (89%)	494 (91.48%)
Presence of comorbidity, p-value = 0.145							
No comorbidity	17 (100%)	15 (71.43%)	334 (89.30%)	17 (94.44%)	10 (100%)	85 (85%)	478 (88.52%)
Presence of one condition	00 (00%)	05 (23.81%)	27 (7.22%)	01 (5.56%)	00 (00%)	11 (11%)	44 (8.15%)
Presence of two or more conditions	00 (00%)	01 (4.76%)	13 (3.48%)	00 (00%)	00 (00%)	04 (4%)	18 (3.33%)
Initial presenting symptoms							
Fever	08 (57.14%)	16 (80%)	277 (80.06%)	13 (72.22%)	05 (71.43%)	65 (73.03%)	384 (77.73%)
Cough	05 (35.71%)	13 (65%)	146 (42.20%)	07 (38.89%)	04 (57.14%)	34 (38.20%)	209 (42.31%)
Fatigue/Weakness	00 (00%)	02 (10%)	78 (22.54%)	02 (11.11%)	01 (14.29%)	10 (11.24%)	93 (18.83%)
Myalgia	03 (21.43%)	00 (00%)	74 (21.39%)	01 (5.56%)	02 (28.57%)	13 (14.61%)	93 (18.83%)
Cold and rhinorrhea	07 (50%)	06 (30%)	163 (47.11%)	11 (61.11%)	03 (42.86%)	47 (52.81%)	237 (47.98%)
Headache	00 (00%)	02 (10%)	26 (7.51%)	00 (00%)	02 (28.57%)	10 (11.24%)	40 (8.10%)
Diarrhea	00 (00%)	00 (00%)	05 (1.45%)	02 (11.11%)	00 (00%)	03 (3.37%)	10 (2.02%)
Breathlessness	00 (00%)	02 (10%)	22 (6.36%)	00 (00%)	01 (14.29%)	06 (6.74%)	31 (6.28%)
Vomiting	00 (00%)	00 (00%)	09 (2.60%)	00 (00%)	00 (00%)	02 (2.25%)	11 (2.42%)
Sore throat	01 (7.14%)	01 (5%)	15 (4.34%)	01 (5.56%)	02 (28.57%)	16 (17.98%)	36 (7.29%)
Skin rash	00 (00%)	00 (00%)	03 (0.87%)	00 (00%)	00 (00%)	00 (00%)	03 (0.61%)
Chest pain	00 (00%)	00 (00%)	01 (0.29%)	00 (00%)	00 (00%)	00 (00%)	01 (0.20%)
Loss of taste	00 (00%)	00 (00%)	02 (0.58%)	00 (00%)	00 (00%)	02 (2.25%)	04 (0.81%)
Loss of smell	00 (00%)	00 (00%)	02 (0.58%)	00 (00%)	00 (00%)	01 (1.12%)	03 (0.61%)
Type of quarantine, p-value = 0.163							
Home quarantine	16 (94.12%)	14 (66.67%)	280 (74.87%)	15 (83.33%)	09 (90%)	80 (80%)	414 (76.67%)
Institutional quarantine/required hospitalization	01 (5.88%)	07 (33.33%)	94 (25.13%)	03 (16.67%)	01 (10%)	20 (20%)	126 (23.33%)
Treatment, p-value = 0.067							
No treatment taken	03 (17.65%)	00 (00%)	10 (2.67%)	00 (00%)	00 (00%)	04 (4%)	17 (3.15%)
Need for conservative treatment	13 (76.47%)	17 (80.95%)	330 (88.24%)	18 (100%)	10 (100%)	87 (87%)	475 (87.96%)
Need for supplemental oxygen	01 (5.88%)	04 (19.05%)	27 (7.22%)	00 (00%)	00 (00%)	05 (5%)	37 (6.85%)
Low-flow oxygen	01 (100%)	03 (75%)	23 (85.18%)	00 (00%)	00 (00%)	04 (80%)	31 (83.78%)
Intubation	00 (00%)	01 (25%)	04 (14.82%)	00 (00%)	00 (00%)	01 (20%)	06 (16.22%)
Need for antiviral agents/steroids or immunomodulatory drugs	00 (00%)	00 (00%)	07 (1.87%)	00 (00%)	00 (00%)	04 (4%)	11 (2.04%)
The outcome of disease, p-value = 0.495							
Survived	17 (100%)	21 (100%)	362 (96.79%)	18 (100%)	10 (100%)	96 (96%)	524 (97.04%)
Dead	00 (00%)	00 (00%)	12 (3.21%)	00 (00%)	00 (00%)	04 (4%)	16 (2.96%)
Vaccination status, p-value = 0.181							
Vaccinated with at least one dose	13 (76.47%)	18 (85.71%)	341 (91.18%)	15 (83.33%)	10 (100%)	94 (94%)	491 (90.93%)
Single dose	00 (00%)	00 (00%)	15 (4.40%)	00 (00%)	00 (00%)	02 (2.13%)	17 (3.46%)
Two doses	11 (84.62%)	16 (88.89%)	238 (69.79%)	11 (73.33%)	06 (60%)	68 (72.34%)	329 (73.27%)
Booster dose	02 (15.38%)	02 (11.11%)	88 (25.81%)	04 (26.67%)	04 (40%)	24 (25.53%)	104 (23.16%)
Not vaccinated	04 (23.53%)	03 (14.29%)	28 (7.49%)	03 (16.67%)	00 (00%)	03 (3%)	41 (7.59%)
Data not available	00 (00%)	00 (00%)	05 (1.34%)	00 (00%)	00 (00%)	03 (3%)	08 (1.48%)

**Figure 6:**
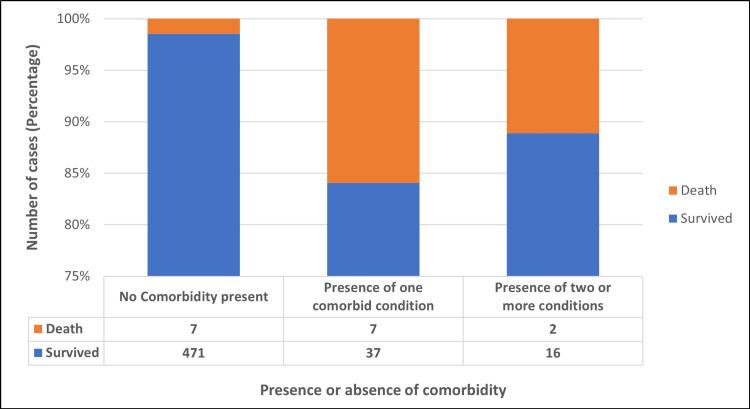
Presence or absence of comorbidity versus the outcome of the disease.

Most cases were symptomatic (91.48%) with mild symptoms. Fever (77.73%) was the most common symptom across all variants, followed by cold and rhinorrhea (47.98%), cough (42.31%), and myalgia and fatigue (18.83%). Of the 540 cases, 478 (88.52%) cases confirmed the presence of no comorbidity. Among those with one or more comorbid conditions, diabetes mellitus was the most common condition reported (51.61%), followed by hypertension (50%), carcinoma (8.06%), coronary heart disease (6.45%), liver and kidney disease (4.84%), asthma (4.84%), and tuberculosis and arthritis (3.23% each). There was a statistically significant difference between the absence or presence of comorbidity and the outcome of disease (p= <0.001) (Figure 6).

**Figure 7:**
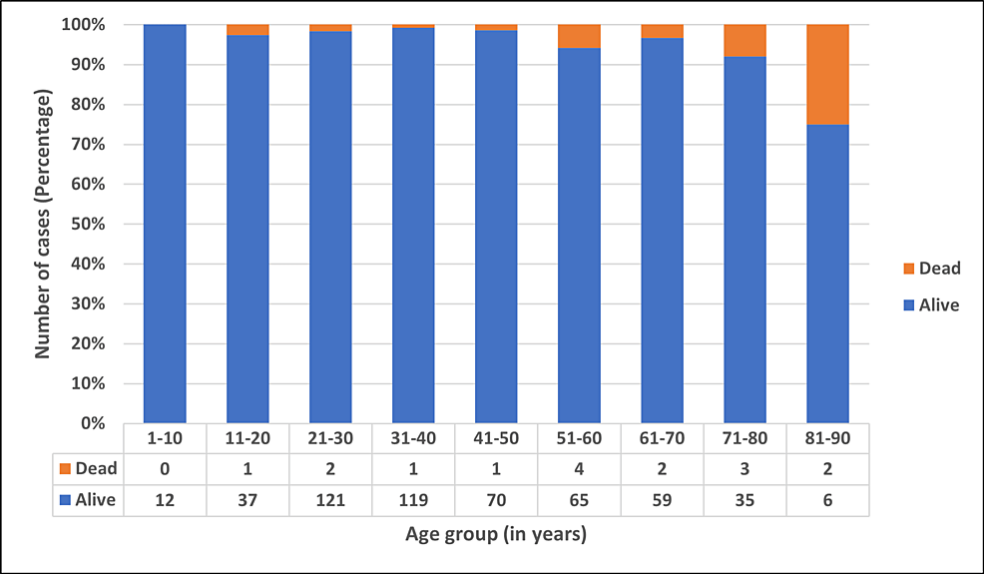
Age-wise distribution of survived and dead cases.

There were 414 (76.67%) cases who required home isolation, and 126 (23.33%) cases were institutionally quarantined/hospitalized. Of the 540 cases, 3.15% recovered without any treatment, 87.96% recovered with supportive treatment, 6.67% required oxygen therapy, and 2.22% were given antiviral treatment. There were 16 (2.96%) cases who succumbed to the disease, and the rest (97.04%) survived. Figure 7 depicts the age-wise distribution of alive and dead cases.

**Figure 8:**
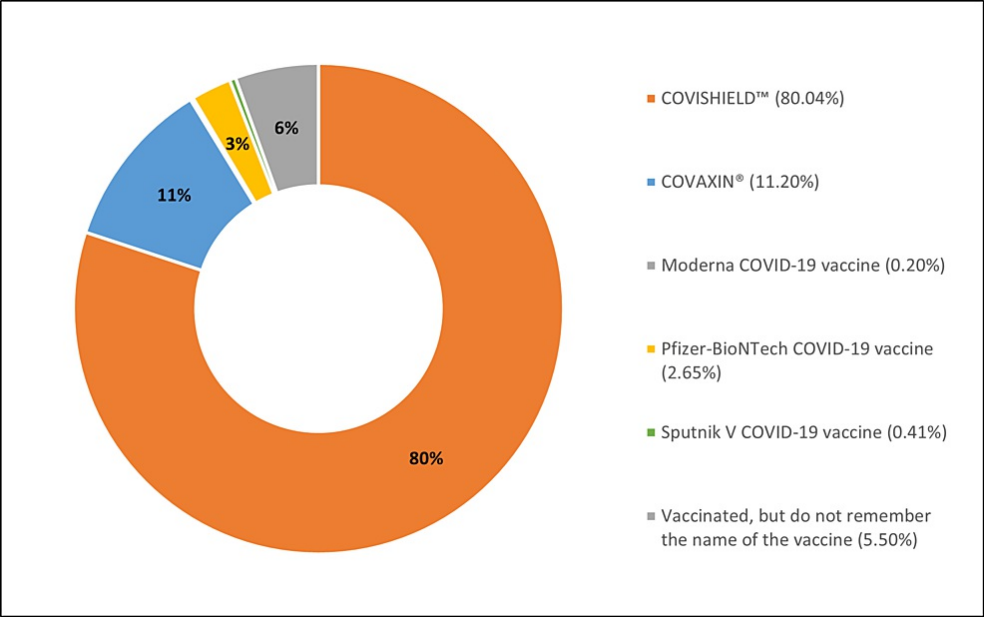
Type of vaccine administered to the study population.

Among the 540 cases, 491 (90.93%) were vaccinated with at least a single dose of the COVID-19 vaccine, 41 (7.59%) were unvaccinated, and for eight (1.48%) cases, vaccination data was not available. Figure 8 enumerates the type of vaccine administered to the study population, with Covishield™ (ChAdOx1 nCoV-19 Corona Virus Vaccine) (80.04%) being the most common vaccine, followed by Covaxin® (BBV152A- a whole inactivated virus-based COVID-19 vaccine) (11.20%).

**Figure 9:**
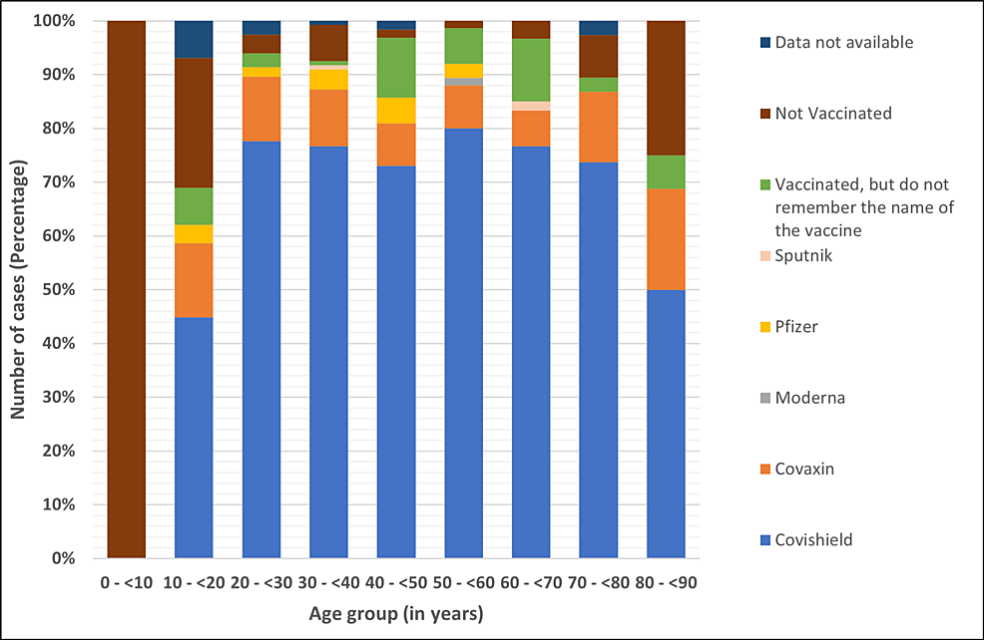
Vaccination status by age groups.

Figure 9 describes the vaccination status of the study population for the age groups. Most unvaccinated individuals were in the age group of 0 to 10 years (25%) and were not offered vaccination as a part of the vaccination policy in the country.

**Figure 10:**
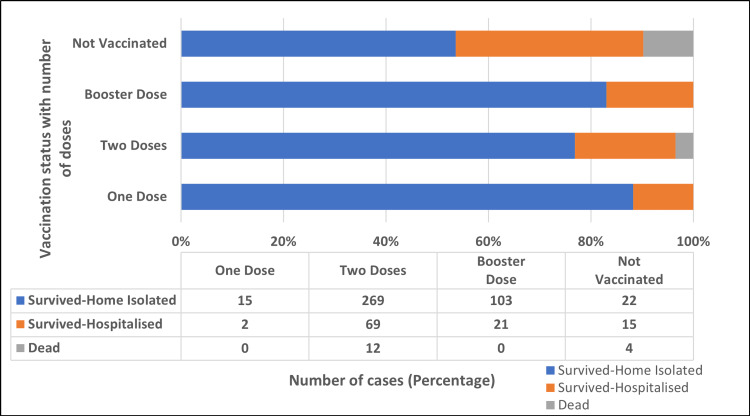
Impact of vaccination on the survival of cases.

Figure 10 shows the impact of vaccination on the survival of cases. Out of 532 cases with available vaccination data, 491 (92.29%) were vaccinated with at least one dose of the vaccine, of whom 479 (97.56%) survived, and 12 (2.44%) died. Similarly, out of 41 (7.71%) cases who were not vaccinated, 37 (90.24%) survived, and four (9.76%) died. The impact of vaccination on the disease outcome was statistically significant (p = 0.001).

Clinical characteristics of XBB.1.5 SARS-CoV-2 variant detected during the study

Of the seven XBB.1.5 cases, history was available for six cases. Three out of six cases (50%) had a history of previous infection. All six cases had mild symptoms, with cold and rhinorrhea (100%) being the most common symptom, followed by sore throat (50%), fever (66.67%), and cough (33.33%). None required hospitalization or supplemental oxygen, and all recovered with conservative treatment at home. Out of the six cases, four had a history of international travel to the United States and Germany, one to Gujarat, and one had contact with a positive person with a history of travel to the United States. All six cases were vaccinated with at least one dose of vaccine, of which two (33.33%) cases had received two doses, and four (66.67%) had received booster doses. No death was reported among the detected XBB.1.5 cases.

## Discussion

**Figure 11:**
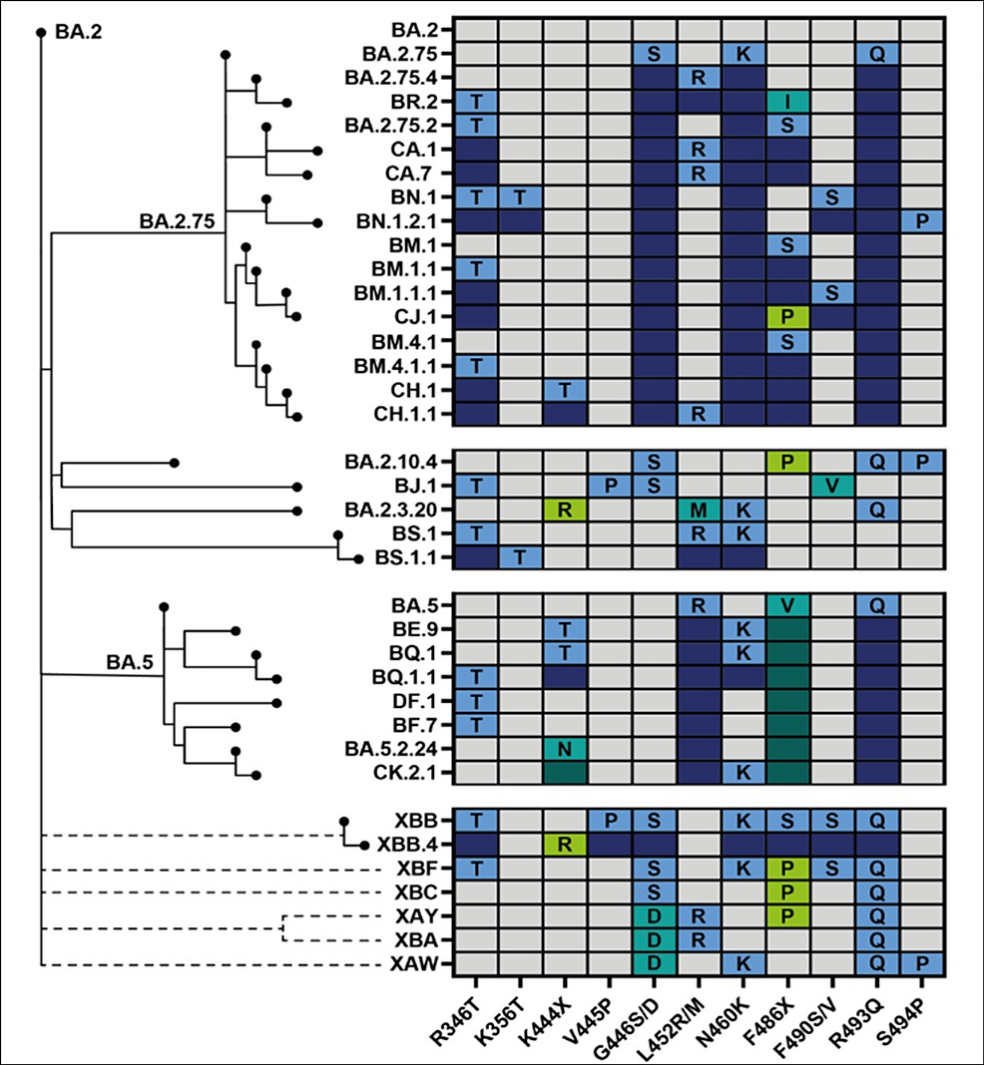
Phylogenetic relatedness and convergent evolution of newer SARS-CoV-2 lineages. This figure is taken from SARS-CoV-2 evolution, post-Omicron, available from https://virological.org/t/sars-cov-2-evolution-post-omicron/911 [11].

**Figure 12:**
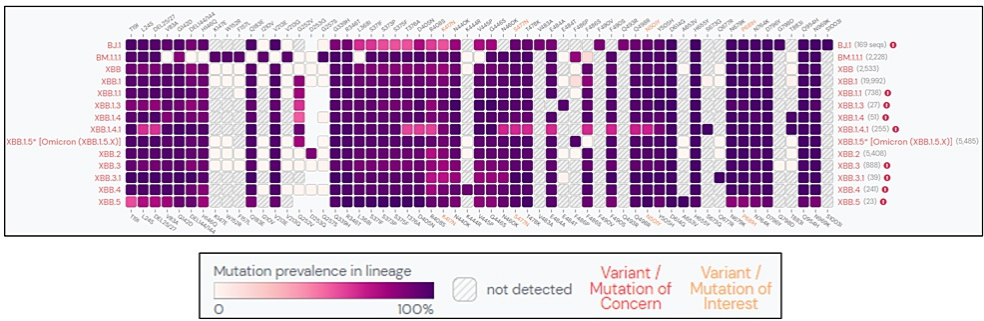
Mutation prevalence across lineages BJ.1, BM.1.1.1, and XBB* (mutations with >75% prevalence in at least one lineage). This figure is generated and taken from Outbreak.info. Data represented is as of January 24, 2023 [15].

The SARS-CoV-2 virus is known for its unique evolutionary characteristic compared to other respiratory viruses (Figure 11). In 2022, saltation or *variant* evolution gave rise to *second-generation variants* evolving from a BA.2 lineage background that have numerous non-synonymous mutations, concentrated in the N-terminal domain (NTD) and the receptor binding domain (RBD) of the spike protein. A few examples include BA.2.75, BA.2.10.4, BJ.1, BS.1, BA.2.3.20, BA.2.83, BP.1, and DD.1, of which BA.2.75 is the most widespread second-generation BA.2 variant [11]. Unlike previous dominant lineages, BA.5 accumulated potent antigenic mutations in a step-wise manner as a result of antigenic drift. The most rapidly growing sublineage of BA.5 is BQ.1, of which BQ.1.1 is the largest containing three further antigenic mutations [11]. SARS-CoV-2 virus, being a coronavirus, is prone to inter-lineage recombination. It generally occurs when a wave declines and a new variant emerges [12]. The resultant recombinant variant possesses unique advantageous properties from both parents [13]. There are 62 recombinant lineages designated by Pangolin (as of December 2022) [14], denoted by a prefix X, of which XBB is the most widespread inter-lineage recombinant to date [11]. XBB is a recombination of BJ.1 (5' part of XBB genome) and BM.1.1.1 (3' part of XBB genome) with a breakpoint between 22,897 and 22,941 positions in the RBD of the spike protein (corresponding to amino acid positions 445-460) [11, 6]. XBB.1.5, a rapidly growing subvariant of the XBB.1 variant, carries an additional mutation F486P in its spike protein, a rare two-nucleotide substitution compared to its parent strain [15]. Figure 12 depicts the mutation prevalence across lineages BJ.1 and BM.1.1.1 and XBB* variant [16]. XBB is shown to have substantially higher viral fitness (Re) than its parental lineages, making it the first documented example of a SARS-CoV-2 variant with increased fitness through recombination [17].

**Figure 13:**
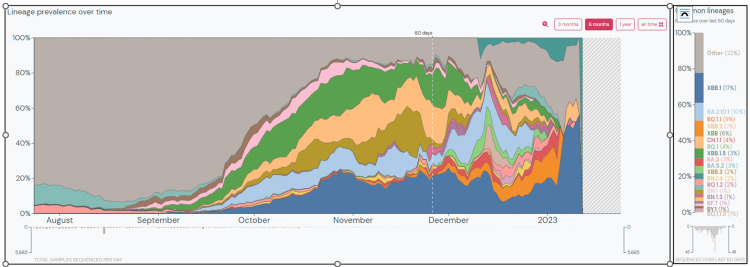
Prevalence of common lineages in India over the last 60 days. This graph is generated and taken from Outbreak.info. Data represented is as of January 24, 2023 [23].

In the present study, between July 10, 2022, and January 12, 2023, BA.2.75* was the predominant Omicron variant. In India, as of January 24, 2023, the apparent cumulative prevalence of BA.2.75*, XBB*, BA.2.38*, BA.2.10*, BA.5*, BQ.1*, and XBB.1.5 is 14%, 15.68%, 7%, 17%, 4%, 3%, and 5%, respectively [5,9,18-22]. Over the last 60 days, XBB.1 is the dominant lineage (17%) in the country (Figure 13) [23]. XBB has also spread to countries like Oman, Dominican Republic, Malaysia, Iraq, Singapore, Indonesia, and Uganda, with a prevalence of 25% to 93% in the last 60 days as of January 24, 2023 [8]. On the contrary, BA.5 and its descendant lineages dominate globally, with about 70.5% of sequences submitted to GISAID between December 26, 2022, to January 1, 2023 [2]. However, the prevalence of BA.5 is decreasing globally with a rise in BA.2 descendant lineages, particularly BA.2.75*. The worldwide apparent cumulative prevalence of BQ.1*, BA.5*, BA.2.75*, XBB*, and XBB.1.5 is 2%, 14%, 1%, 2.48%, and 1%, respectively (as of January 24, 2023) [5,9,18,19,22]. The regional difference in the prevalence of XBB and BQ.1 lineages, XBB being more dominant in the eastern hemisphere and BQ.1 in the western hemisphere, may be due to the proximity of these regions to places where these lineages originated [17]. However, the recently detected XBB.1.5 variant is shown to have a growth advantage over other circulating Omicron sub-lineages and has already been reported from 46 countries [9]. As of January 24, 2023, 9,173 sequences have been deposited on GISAID, with the majority of sequences from the United States (74.63%), United Kingdom (9.55%), Canada (3.02%), Austria (2.57%), and Denmark (2.02%) [24].

The most remarkable feature of XBB is its profound resistance to humoral immunity induced by infections with prior Omicron variants. In-vitro studies have shown that XBB exhibited 30-fold and 13-fold resistance to BA.2 and BA.5 infection sera, respectively [17]. Similar findings were reported by Wang et al., where XBB was ~63-fold and ~49-fold more resistant to neutralization than BA.2 and BA.4/5, respectively [6]. Several NTD and RBD spike mutations such as V83A, Y144del, Q183E, R346T, L368I, V445P, F486S, and F490S cooperatively contribute to resistance against humoral immunity induced by BA.2 infections. Similarly, two mutations, Y144del and G446S, were suggested to contribute to resistance against humoral immunity induced by BA.5 infections. The humoral immune-escape property of XBB.1.5 was comparable to XBB.1 [15]. Spike mutations in the RBD (R346T, L368I, and N460K) and NTD (V83A) are also responsible for increased ACE2 binding affinity, viral infectivity, and fusogenicity of XBB compared to BA.2, BA.5, and BA.2.75 Omicron variants [17]. Deep mutational scanning studies have shown that due to the presence of additional Ser486Pro mutation in the spike protein, XBB.1.5 had a stronger binding affinity to ACE2 receptors than XBB.1, thereby explaining its significant growth advantage over XBB.1 [15]. XBB-infected hamster sera showed a remarkable antiviral effect against XBB only, suggesting XBB is antigenically distinct from other Omicron subvariants [17].

Several clinically authorized therapeutic monoclonal antibodies (mAbs) such as bamlanivimab, etesevimab, imdevimab, casirivimab, tixagevimab, cilgavimab, and sotrovimab have been rendered ineffective by previous SARS-CoV-2 variants, leaving bebtelovimab as the only monoclonal antibody active against the circulating strains. However, due to mutations such as N460K, F486S, R346T, V455P, G446S and F490S, XBB, and its descendant XBB.1 are pan-resistant to RBD class I, II, and III antibodies [6]. Moreover, bebtelovimab (LY-CoV1404) and Evusheld (a combination of COV2-2196 and COV2-2130) were found to be inactive against XBB/XBB.1 [6] and XBB.1.5 [15]. Although XBB.1.5 escapes neutralizing antibody responses, a study by Lasrado et al. has shown that the cross-reactive T-cell responses may continue to protect against severe disease [25]. Therefore, due to immune-escape-associated and infectivity-enhancing mutations, the XBB* and XBB.1.5 variants can eventually spread worldwide [17].

The present study suggests that the pathogenicity of XBB is comparable to that of other Omicron variants. These findings are consistent with an in-vivo study in hamsters where the XBB variant was less pathogenic than the Delta variant and had a comparable pulmonary function and viral RNA load to BA.2.75-infected hamsters. The intrinsic pathogenicity of the XBB variant and its efficiency of infecting lungs was comparable to or even lower than the BA.2.75 [17]. Similarly, the seven XBB.1.5 cases detected had mild symptoms. However, it remains unclear whether the intrinsic pathogenicity of the virus or the immunity from vaccination and previous infection is responsible for mild cases in India. More clinical data from other clinical settings with different levels of immunity will help understand the behavior of the XBB* variant.

## Conclusions

The present study describes the clinical characteristics and outcomes of the major SARS-CoV-2 variants circulating in the community in Maharashtra. While it is encouraging that the infection caused by the SARS-CoV-2 XBB recombinant variant tends to be less severe and similar to that of other Omicron subvariants, the fact that this variant is more transmissible and immune-evasive is a cause of concern. In addition to the ongoing surveillance efforts to track the spread of new variants, our findings emphasize the importance and the need to monitor the clinical severity of infections caused by new variants. It also shows the impact of vaccination on the survival of infected cases. Such findings are essential for making decisions about deploying interventions and preparing healthcare systems to respond to outbreaks.
